# Human Listeriosis Caused by *Listeria ivanovii*

**DOI:** 10.3201/eid1601.091155

**Published:** 2010-01

**Authors:** Christelle Guillet, Olivier Join-Lambert, Alban Le Monnier, Alexandre Leclercq, Frédéric Mechaï, Marie-France Mamzer-Bruneel, Magdalena K. Bielecka, Mariela Scortti, Olivier Disson, Patrick Berche, José Vazquez-Boland, Olivier Lortholary, Marc Lecuit

**Affiliations:** Université Paris Descartes, Paris, France (C. Guillet, O. Join-Lambert, F. Mechaï, M.-F. Mamzer-Bruneel, P. Berche, O. Lortholary, M. Lecuit); Hôpital Necker–Enfants Malades, Paris (C. Guillet, O. Join-Lambert, F. Mechaï, M.-F. Mamzer-Bruneel, P. Berche, O. Lortholary, M. Lecuit); Institut Pasteur, Paris (A. Le Monnier, A. Leclercq, O. Disson, M. Lecuit); University of Edinburgh, Edinburgh, Scotland, UK (M. K. Bielecka, M. Scortti, J. Vazquez-Boland); Universidad Complutense de Madrid, Madrid, Spain (M. Scortti); Institut National de la Santé et de la Recherche Médicale, Paris (O. Disson, M. Lecuit); and Universidad de Léon, Léon, Spain (J. Vazquez-Boland); 1 These authors contributed equally to this article.

**Keywords:** Listeria ivanovii, gastroenteritis, bacteremia, kidney transplantation, bacteria, dispatch

## Abstract

Two species of *Listeria* are pathogenic; *L. monocytogenes* infects humans and animals, and *L. ivanovii* has been considered to infect ruminants only. We report *L. ivanovii*–associated gastroenteritis and bacteremia in a man. This isolate was indistinguishable from prototypic ruminant strains. *L. ivanovii* is thus an enteric opportunistic human pathogen.

The genus *Listeria* contains 2 pathogenic species, *L. monocytogenes* and *L. ivanovii* (*1*). They both invade host cells, replicate in the cytosol after phagosomal escape, and spread from cell to cell by polymerizing actin. These mechanisms correlate with the presence in each species of genetic determinants called the *inlAB* internalization locus, the LIPI-1 intracellular survival pathogenicity island, and the *hpt* intracellular growth locus (*2*). However, each species appears to infect different hosts: *L. monocytogenes* infects humans and ruminants, whereas *L. ivanovii* is thought to infect ruminants only (*2*). *L. ivanovii* have been previously isolated, although rarely, from infected humans, indicating pathogenic potential for humans (Table). We report a case of *L. ivanovii* infection in a man with a kidney transplant. The ecology of *L. ivanovii* suggests that the rarity of human listeriosis due to this species reflects not only host tropism factors but also the rare occurrence of this species in the environment, compared with *L. monocytogenes*.

**Table:** Reported human cases of *Listeria ivanovii* infection

Clinical condition	Sex	Underlying condition	Year reported (reference)
Unknown*	Unknown*	Unknown*	1971 (*3*)
Uterine discharge	F	Pregnancy	1985 (*4*)
Mesenteric adenitis	Unknown	Unknown	1985 (*4*)
Stillbirth	F	Pregnancy	1990 (*5*)
Bacteremia	M	AIDS, lymphoma	1994 (*6*)
Bacteremia	M	Substance abuse	1994 (*7*)
Bacteremia	M	Hepatic carcinoma	2006 (*8*)
Gastroenteritis, bacteremia	M	Immunosuppression†	2007 (this study)

*Published as *Listeria monocytogenes* serovar 5 (pre-1986 designation of *L. ivanovii*). †Polycystic kidney disease (which led to chronic renal failure and renal transplantation), chronic hepatitis C.

## The Case

In January 2007, a 55-year-old man was hospitalized in Paris, France, with a 3-week history of nonbloody diarrhea, vomiting, dehydration, and low-grade fever. Medical history included renal transplantation for chronic renal failure and chronic hepatitis C. Immunosuppressive regimen included mycophenolate mofetil, tacrolimus, and prednisone. At the time of admission, his temperature was 37.8°C and he had moderate and painless abdominal distension. Laboratory values were 5.9 × 10^9^/L leukocytes, 0.4 × 10^9^/L lymphocytes, 9.7 g/dL hemoglobin, 137,000/mL platelets, 470 µmol/L creatinine, and <5 mg/L serum C-reactive protein. Liver tests were within normal limits except γ-glutamyltranferase, which was increased (244 U/L; reference <50 U/L).

Blood cultures yielded coryneform gram-positive rods with intensely β-hemolytic colonies; catalase and esculin hydrolysis test results were positive, consistent with *Listeria* spp. (*1*). Because listeriosis was suspected, intravenous amoxicillin and gentamicin therapy was initiated. Cerebrospinal fluid showed no abnormalities by direct examination or culture. Semiquantitative aerobic fecal culture showed the same coryneform gram-positive rods (10^6^ CFU/g). The API Coryne biochemical test (bioMérieux, Marcy l’Étoile, France) identified blood and fecal isolates as *Listeria* spp. Fecal specimens were negative for *Salmonella*, *Shigella*, *Yersinia*, and *Campylobacter* spp. After 7 days, intravenous treatment was switched to oral amoxicillin for 2 weeks. The patient’s condition rapidly improved, and control fecal cultures were negative.

The 3 isolates from blood and 1 from feces were referred to the French National Reference Centre for *Listeria* (Institut Pasteur, Paris, France). All were identified as *L. ivanovii* subsp. *ivanovii* and belonged to *L. ivanovii*–specific serovar 5. They showed identical profiles by pulsed-field gel electrophoresis (Figure, panel A). Agar diffusion test results were as expected for *Listeria* spp.: susceptible to amoxicillin and gentamicin; resistant to third-generation cephalosporins, clindamycin, and aztreonam (*2*). Contrary to *L. monocytogenes*, which is naturally resistant to fosfomycin in vitro (*9*), all isolates were susceptible to fosfomycin in vitro, as previously reported (*2*).

**Figure:**
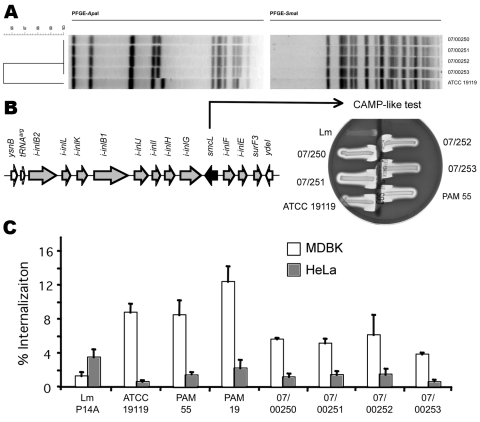
Characterization of the *Listeria ivanovii* subsp. *ivanovii* isolates from a 55-year-old man with gastroenteritis and bacteremia. A) The 4 isolates, 07/00250, 07/00251, and 07/00252 from blood, and 07/00253 from feces, were analyzed by pulsed-field gel electrophoresis (PFGE) with *Apa*I and *Sma*I restriction enzymes (*9*). The *L. ivanovii* subsp. *ivanovii* type strain American Type Culture Collection (ATCC) 19119 was used as control. Profiles were compared according to band positions by using the Dice coefficient and were clustered by using unweighted pair–group method averages. Criterion of dissimilarity = 1 band difference (maximum position tolerance 1.5%). B) *L. ivanovii*–specific virulence locus LIPI-2 and its phenotypic marker (sphingomyelinase production as shown by a CAMP-like test with an indicator strain of *Rhodococcus equi* on sheep blood agar). Left, genetic structure of LIPI-2. Arrowheads indicate positions of the oligonucleotide primers used in the 19 intragenic and intergenic PCRs to map the locus in the isolates; arrows represent genes (those belonging to LIPI-2 are gray, the sphingomyelinase gene is black, and flanking genes from the core listerial genome are white) (*10*). Right, typical shovel-shaped synergistic hemolysis reactions caused by *L. ivanovii* sphingomyelinase in the presence of *R. equi* cholesterol oxidase compared with the negative reaction given by *L. monocytogenes* (Lm). C) Invasion (gentamicin protection) assays in bovine (Madin-Darby bovine kidney) and human (HeLa) epithelial cells. The human isolates were compared with ruminant isolates ATCC 19119, PAM 55, and PAM 19 and with the *L. monocytogenes* strain P14A. Error bars indicate SEM of at least 2 duplicate experiments.

The isolates were compared with prototypic *L. ivanovii* strains from sheep (American Type Culture Collection 19119 type strain, Ivan Ivanov, 1955, PAM 19, Australia) and goats (PAM 55, Spain). We determined the activation status of the virulence gene regulator PrfA. For *L. monocytogenes,* the PrfA-regulated factors are mainly expressed in vivo, but for *L. ivanovii*, they are constitutively overexpressed in vitro (*2**,**11*). Some of these virulence factors have easily detectable phenotypes, such as hemolysis on blood agar, PlcB phospholipase activity on egg yolk agar, and Hpt hexose phosphate transporter activity in acidification test (*2*,*12*). All isolates were phenotypically identical; they produced broad halos of hemolysis and lecithinase reactions and had positive glucose-1-phosphate acidification test results, reflecting the constitutive activation of the PrfA virulence regulon.

PCR mapping was used to test for *L. ivanovii*–specific pathogenicity island LIPI-2 (*13*). LIPI-2 comprises 10 internalin genes and the sphingomyelinase gene *smcL* and is perfectly conserved within *L. ivanovii,* including the distantly related subspecies *londoniensis* (*13*). All intragenic and intergenic PCRs gave identical results for all strains. The phenotypic marker for LIPI-2, *smcL*-encoded sphingomyelinase, was assessed by the synergistic hemolysis (CAMP-like) test (*13*) and was found in all strains (Figure, panel B).

Finally, we performed invasion assays with Madin-Darby bovine kidney (MDBK) cells and HeLa cells (human). Confirming previous observations (*13*), all *L. ivanovii* strains were hyperinvasive in MDBK cells and less invasive in HeLa cells compared with *L. monocytogenes* (Figure, panel C). Invasion assays expressing human E-cadherin or not did not show substantial differences, suggesting that *L. ivanovii* InlA does not interact with E-cadherin, in contrast to *L. monocytogenes* InlA (*6*) (data not shown). The 4 patient isolates showed slightly lower invasion capacity in MDBK cells than did isolates from ruminants but were still hyperinvasive relative to *L. monocytogenes*.

## Conclusions

We found 3 other well-documented cases of *L. ivanovii*–associated human infection (Table) 1 febrile diarrhea (*7*) and 2 bacteremia cases (*8*,*10*). The infections were associated with AIDS, metastatic carcinoma, or substance abuse; 2 patients were >60 years of age. Thus, as for *L. monocytogenes* (*1*), human *L. ivanovii* infection is associated with immunodeficiency, underlying debilitating conditions, or advanced age. In at least 3 other instances, bacteria were found in human samples, 2 in fetoplacental tissue and lochia and 1 in a mesenteric lymph node (*4**,**5*) (Table). The pathologic changes associated with *L. ivanovii* in humans appear similar to those in ruminants, i.e., fetoplacental infections and septicemia (often accompanied by enteritis). Typically, meningoencephalitis is not caused by *L. ivanovii* in ruminants, whereas it is a hallmark of *L. monocytogenes* infection in ruminants and humans (*1*). Lack of central nervous system involvement could be a general characteristic of *L. ivanovii* infection regardless of host species. The specific pathogenic features of *L. ivanovii* may be caused by sequence differences in virulence genes shared with *L. monocytogenes* or by differences in the gene content of these 2 species (*1*,*6*).

These human cases raise questions about the supposed specificity of *L. ivanovii* for ruminants. Although the rare occurrence of *L. ivanovii* infections in humans (*3*) could result from lower pathogenicity for humans, it may reflect ecologic characteristics of the species. *L. ivanovii* is isolated only occasionally from animals or environmental sources (*2*,*4**,**5*), suggesting a limited distribution in nature, including in food. Therefore, the few human cases of *L. ivanovii* infection reported might correspond to what would be proportionally expected for a species with such sporadic occurrence.

That gastroenteritis preceded bacteremia and that the same isolates were found in the feces strongly indicate a foodborne infection in the patient reported here and that *L. ivanovii* causes gastroenteritis in humans, as reported for *L. monocytogenes* (*14*). Days before onset of gastroenteritis, the patient had eaten artisanal goat cheese made from raw milk. Unfortunately, no cheese sample was available for bacteriologic investigation. Although the portal of entry of *L. ivanovii* has not been formally established, *L. ivanovii* infection in ruminants is associated with eating spoiled silage or hay, as happens with *L. monocytogenes*, suggesting foodborne origin. *L. ivanovii* has been isolated from food, including goat milk (*15*).

Simultaneous detection of *L. ivanovii* in the feces and blood of a human, together with previous association between *L. ivanovii* and human mesenteric adenitis (*5*), suggests that these bacteria can cross the intestinal barrier in humans, cause gastroenteritis, and disseminate into the bloodstream. Although *L. monocytogenes* are by far the leading cause of human listeriosis, our report shows that *L. ivanovii* can also cause bacteremia in immunocompromised, debilitated patients.
